# 2262. Evaluation of Ceftriaxone Versus Cefazolin as a Surrogate Marker for Cefpodoxime Susceptibility in Enterobacterales Isolates

**DOI:** 10.1093/ofid/ofad500.1884

**Published:** 2023-11-27

**Authors:** Kaitlyn Lambert, Ryan Demkowicz, Douglas Slain, Amanda Murray, Catessa A Howard

**Affiliations:** WVU Medicine, Morgantown, West Virginia; West Virginia University, Morgantown, West Virginia; West Virginia University, Morgantown, West Virginia; WVU Medicine, Morgantown, West Virginia; West Virginia University Medicine, Morgantown, West Virginia

## Abstract

**Background:**

Cefpodoxime is increasingly being explored as an option for intravenous-to-oral step-down therapy in Enterobacterales infections, but it is rarely included in routine susceptibility testing. The Clinical and Laboratory Standards Institute (CLSI) states that cefpodoxime susceptibility among urinary isolates can be inferred from the result for cefazolin. However, cefazolin resistance may overcall resistance to cefpodoxime. Studies directly comparing ceftriaxone, cefazolin, and cefpodoxine susceptibilities are lacking. The purpose of this study was to determine correlation of ceftriaxone or cefazolin susceptibilities as a surrogate marker for cefpodoxime susceptibility in Enterobacterales.

**Methods:**

The study was conducted on clinical isolates of *E. coli*, *Klebsiella* spp., and *Proteus* spp. from urine, blood, respiratory, surgical, or wound cultures. Susceptibility testing was conducted by manual Kirby Bauer disk diffusion for cefpodoxime, ceftriaxone, and cefazolin (in duplicate) following standard procedures. The VITEK 2 automated system was used to adjudicate discrepancies between duplicates. Breakpoints from the current CLSI M100 were used to assign categories (S, R, I) for the different methods of susceptibility testing. Categorical agreement, very major, major, and minor error rates between ceftriaxone or cefazolin and cefpodoxime were calculated.

**Results:**

A total of 88 clinical isolates were evaluated. The categorical agreement rate was 64% for cefazolin and 97% for ceftriaxone (*P* =0.0001). The major error rate was 15% (11/74) for cefazolin and 0% for ceftriaxone. The very major error rate was 7% (1/14) for cefazolin and 21% (3/14) for ceftriaxone. The minor error rate was 22% (19/88) for cefazolin and 0% for ceftriaxone.

Isolate Characteristics

**Figure:**
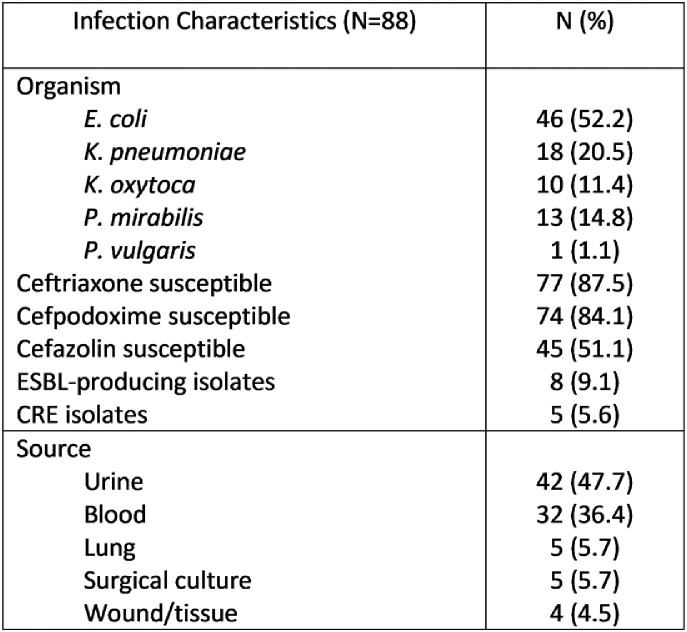

**Conclusion:**

Ceftriaxone appears to be a better surrogate marker for predicting cefpodoxime susceptibility, when compared to cefazolin. In addition, ceftriaxone demonstrated lower major and minor error rates.

**Disclosures:**

**All Authors**: No reported disclosures

